# The Mechanism of *Bacteroides fragilis* Toxin Contributes to Colon Cancer Formation

**DOI:** 10.21315/mjms2020.27.4.2

**Published:** 2020-08-19

**Authors:** Wai Teng Cheng, Haresh Kumar Kantilal, Fabian Davamani

**Affiliations:** 1 Applied Biomedical Sciences and Biotechnology, School of Health Sciences, International Medical University, Kuala Lumpur, Malaysia; 2 Division of Pathology, School of Medicine, International Medical University, Kuala Lumpur, Malaysia

**Keywords:** Bacteroides fragilis, colon cancer, STAT3 pathway, Bacteroides fragilis toxin, inflammation

## Abstract

The *Bacteroides fragilis* (*B. fragilis*) produce biofilm for colonisation in the intestinal tract can cause a series of inflammatory reactions due to *B. fragilis* toxin (BFT) which can lead to chronic intestinal inflammation and tissue injury and play a crucial role leading to colorectal cancer (CRC). The enterotoxigenic *B. fragilis* (ETBF) forms biofilm and produce toxin and play a role in CRC, whereas the non-toxigenic *B. fragilis* (NTBF) does not produce toxin. The ETBF triggers the expression of cyclooxygenase (COX)-2 that releases PGE2 for inducing inflammation and control cell proliferation. From chronic intestinal inflammation to cancer development, it involves signal transducers and activators of transcription (STAT)3 activation. STAT3 activates by the interaction between epithelial cells and BFT. Thus, regulatory T-cell (Tregs) will activates and reduce interleukin (IL)-2 amount. As the level of IL-2 drops, T-helper (T_h_17) cells are generated leading to increase in IL-17 levels. IL-17 is implicated in early intestinal inflammation and promotes cancer cell survival and proliferation and consequently triggers IL-6 production that activate STAT3 pathway. Additionally, BFT degrades E-cadherin, hence alteration of signalling pathways can upregulate spermine oxidase leading to cell morphology and promote carcinogenesis and irreversible DNA damage. Patient with familial adenomatous polyposis (FAP) disease displays a high level of tumour load in the colon. This disease is caused by germline mutation of the *adenomatous polyposis coli* (*APC*) gene that increases bacterial adherence to the mucosa layer. Mutated-*APC* gene genotype with ETBF increases the chances of CRC development. Therefore, the colonisation of the ETBF in the intestinal tract depicts tumour aetiology can result in risk of hostility and effect on human health.

## Introduction

Bacteroides species are non-spore forming, anaerobe and gram-negative bacteria. There are more than 20 different species of Bacteroides. These bacteria act as normal flora in the intestine to maintain healthy intestinal microflora in humans. *Bacteroides fragilis* (*B. fragilis*) has two classes: non-toxigenic *B. fragilis* (NTBF) and enterotoxigenic *B. fragilis* (ETBF) (1). The differences between NTBF and ETBF are the presence of *B. fragilis* toxin (*bft*) gene and its ability to produce biofilm. BFT product is a 20 kDa zinc-dependent metalloprotease toxin, also known as fragilysin or BFT (1–3). BFT plays an important role in intestinal inflammation and tissue injury by damaging the tight junction and increasing intestinal permeability. Furthermore, it has been proven that tissue inflammation and injury promote cancer formation (1, 4). Simultaneously, the biofilm produced by *B. fragilis* induces carcinogenesis. Fortunately, only ETBF encompasses *bft* and can produce biofilms. Hence, NTBF does not harm the intestinal tract (5).

In the United States, colorectal cancer (CRC) is the third most common cancer in both genders. It is also the second most common cancer-related death, especially for older patients who are ≥ 60 years old. In 2013, the American Cancer Society stated that there were 102,480 new cases of CRCs that led to the death of 50,830 people. Moreover, CRC is the fourth leading cancer resulting in deaths worldwide. Inflammatory bowel disease (IBD) and genetic mutations are factors predisposing an individual towards colon cancer; this indicates that CRC has a high mortality rate (6–8).

Microbes are capable in promoting cancer development through several routes such as activation of chronic inflammation, alteration of tumour microenvironment and production of toxins that damage DNA (9). When there is chronic ETBF colonisation in the intestine, it stimulates chronic intestinal inflammation, triggering signal transducers and activators of transcription 3 (STAT3) activation, which contributes to interleukin (IL)-17 production. IL-17 is involved in colon inflammation. BFT produced by ETBF causes the alteration of signalling pathways and production of reactive oxygen species (ROS) that leads to DNA damage and cleavage of E-cadherin (3, 10). In the below review, we have provided a general information regarding BFT produced by ETBF, triggering CRC development.

## Literature Review

### Colon Cancer Associated with Microbes

In the human gastrointestinal tract, there are nearly 100 trillion microbes, out of which 30% make up normal flora in the intestine. Meanwhile, the normal flora is characterised into beneficial and harmful microbes. Beneficial microbes promote nutrition, including production of vitamins in the intestine, and prevent disease formation. However, harmful microbes produce toxin and carcinogenic substances in the intestine. These harmful substances may cause cancer (11). There are many types of bacteria that stimulate a variety of cancer formation through their respective site of inflammation (12), e.g. bacteria, such as *Enterococcus faecalis* (*E. faecalis*), colibactin-producing *Escherichia coli* (*E. coli*) and ETBF are involved in CRC development. However, the mechanisms between each bacterium in contributing to CRC formation are different; for instance, *E. faecalis* damages the DNA through ROS, colibactin-producing *E. coli* produces colibactin that damages the DNA, and ETBF produces BFT that contributes to inflammation and immune-cell infiltration (13).

### Intestinal Dysbiosis, Inflammation and Colon Cancer

Normal flora is advantageous to a person as it maintains intestinal health and gut homeostasis. However, as the bacteria such as ETBF in the gut undergoes dysbiosis, it brings harmful effects to the person. According to Deng et al. (14), a correlation was observed between microbiota imbalance and cancer progression, while Liu et al. (15) claimed that CRC development is associated with intestinal microecology disorder. Imbalance among microbiota leads to bacterial infection that can progress to chronic inflammation. One of the main environmental risk factors contributing to CRC development is chronic intestinal inflammation. Chronic inflammation alters cellular microenvironment, enhances gene mutation, inhibits apoptosis and induces neovascularisation and cell proliferation that causes pre-cancerous conditions, eventually leading cancer (16). Simultaneously, chronic inflammation causes genetic alterations that directly affect the STAT3 pathway and promoting carcinogenesis (17). There are three stages involved in tumour development, namely initiation, promotion and progression (18). During initiation and progression, cancer cells and microbes interact, both producing genetic and inflammatory–immunological factors that are responsible for their survival and replication (19). In tumour progression, tumour cells interact with the inflammatory cells in the tumour microenvironment. These tumour cells secrete inflammatory–immunological factors to attract the inflammatory cells and activate the stromal cells. Simultaneously, both inflammatory and activated stromal cells start to produce various soluble factors, including cytokines, chemokines, growth factors and protease. These soluble factors play an important role in facilitating the growth, differentiation and survival of tumour cells. Hence, it promotes tumour progression and promotion. Additionally, cytokines or microbes promote cancer by changing genetic sequence (18). During gene mutation, epithelial cells replicate rapidly and develop into a hyperplastic epithelium, which progresses into adenomas and then towards adenocarcinomas. Both adenomas and adenocarcinomas affect the growth rate of colonic epithelial cells and improve the cells’ toleration towards apoptosis, and abnormal cells escape from the immune cells. Furthermore, these adenocarcinomas begin to invade submucosa, turning into cancer. When the growth of malignant cells continues, the tumour continues to spread in the colon (13, 20). Thus, carcinogenesis becomes more efficient.

IBD is an example of chronic intestinal inflammation that is associated with ETBF. Pathogenic bacteria are capable of stimulating infection, inflammation and carcinogenesis, whereas the relationship between IBD and CRC is well established (21). Surprisingly, patients with IBD show a high level of immunoglobulin (Ig) G antibodies, IL-6, vascular endothelial growth factor (VEGF) and tumour necrosis factor (TNF). IgG antibodies are responsible for killing bacteria moving into the intestinal lumen (10). Simultaneously, IL-6 and VEGF are responsible for STAT3 activation. IBD is also known as ulcerative colitis (UC) and Crohn’s disease (CD). This chronic intestinal inflammation increases the risk of colitis-associated CRC, the probability of which depends on multiple casual factors, including severity, duration of inflammation in the intestine and gut microbiota imbalance (22–26). Patients with UC or CD have 2–3 folds higher incidence of CRC when compared to healthy individuals. It is also stated that patients with UC and CD have 3.7% and 2.5%, respectively, higher risks of CRC compared to a normal healthy person. This indicates that patients with UC tend to be more susceptible to CRC than those with CD (27, 28). Furthermore, it is evident that the large intestine tends to have a higher risk of CRC compared to the small intestine, which can be attributed to the higher amount of bacteria (29). Simultaneously, people with IBD and CRC have a higher quantity of ETBF in the intestine or stool examination compared to healthy persons (30). Additionally, ETBF are biofilm producers; they can reduce or redistribute E-cadherin in the colonic epithelial cells, trigger the production of IL-6 by epithelial cells, activate STAT3 pathway and enhance cells proliferation at the site of crypt epithelial in normal colon mucosa. This shows that biofilms are associated with the risk of colon cancer development (31).

### COX Enzymes Involved in Inflammation, Carcinogenesis and Biomarker

Chronic inflammation is a principal factor that contributes to carcinogenesis. Prostaglandin is a paracrine hormone that plays an important role in inflammation. Cyclooxygenase (COX) is the rate-limiting enzyme responsible for producing prostaglandins (32). COX-1 and COX-2 are the isoforms of COX enzymes that break down arachidonic acid into prostaglandins. COX-2 plays an important role in maintaining environment for the development of cancer inflammation. COX-2 is normally expressed in epithelial and stromal cells, and the expression level is increased in both inflammation and cancer due to the presence of proinflammatory cytokines. Additionally, BFT triggers colonic epithelial cells to express COX-2 but not COX-1. COX-2 releases prostaglandin E2 (PGE2) that triggers pain and inflammation at the site of tissue injury. Simultaneously, PGE2 controls cell proliferation by binding at the cell receptor and activating oncogenic signalling pathways. Thus, it is proven that COX-2 plays an important role in carcinogenesis and cancer progression by promoting cell proliferation, angiogenesis and cancer stem cell formation; inhibiting cell apoptosis; and heightening metastatic potential through producing PGE2 (3, 17, 18, 33–35).

In certain studies, it is stated that aspirin and non-steroidal anti-inflammatory drugs have the ability to inhibit the activity of COX enzyme, which reduces the inflammatory response; thus, it delays CRC occurrence. Fortunately, COX-1 and COX-2 act as biomarkers for screening purposes. The biomarker is defined as any substance, structure or process that is measurable in the body to determine the incidence of a disease (36). It is commonly detected in circulation and body fluids. COX-1 is present in most cells; thus, it is not a specific biomarker. However, COX-2 is only detected when the inflammation is stimulated by trauma, release of cytokines and stimulation of arachidonate metabolism by a toxin such as BFT. Thus, COX-2 acts as a useful biomarker to detect inflammatory responses (37–39). COX-2 is also a useful biomarker for colorectal carcinogenesis screening. The level of COX-2 biomarker in the blood is dependent upon epithelial cell proliferation, apoptosis inhibition and neoangiogenesis. Patients with CRC have high levels of COX-2 compared to normal individuals (40–42), indicating more aggressive growth rate and higher mortality rate. This suggests that COX-2 expression is correlated to the aggressiveness of growth rate and mortality rate (43).

### ETBF Activates STAT3

ETBF is associated with IBD due to the abnormal regulation of immune response to bacteria. The systemic adaptive immune response is activated to eliminate foreign antigens in the body. This action eventually reduces intestinal mucosal tolerance (10, 44). Although immune cells kill foreign antigens, neutrophils and T_h_17 cells contribute to inflammation and tumourigenesis. Transcription factors are known as STAT protein family comprising seven members. Each STAT protein responds to its specific cytokines. They play an important role in regulating immune responses by controlling T_h_ cell types generation (3, 17, 44, 45); for instance, the activation and generation of T_h_17 cells require transcription factor STAT3 protein (46). The roles of STAT3 protein include promotion of cell proliferation, cell survival, inflammation, cellular transformation, metastasis of cancer, blood vessel formation and tumour-promoting inflammation (45, 47). Moreover, STAT3 is a major intrinsic pathway for cancer inflammation. It induces genes in tumour cells that are responsible for inflammation. Within a tumour cell, it exhibits an overly expressed STAT3 pathway (17).

ETBF has the ability to activate STAT3 rapidly in both colonic epithelial cells and colonic mucosal immune cells through phosphorylation and nuclear translocation. However, STAT3 activation first occurs in colonic mucosal immune cells followed by colonic epithelial cells. To activate STAT3 in immune cells, epithelial cells should respond in the production of cytokines, such as IL-6, IL-10 and IL-23. Besides cytokines, growth factors including VEGF and fibroblast growth factor (FGF2), are also involved in activating STAT3. When ETBF and BFT first interact with colonic epithelial cells, they stimulate early STAT3 activation in colonic mucosal immune cells. This STAT3 activation continuously rises slowly until it reaches the peak level. The peak indicates that ETBF activates the immune system due to barrier dysfunction (10, 48, 49). During ETBF-induced colitis, it activates both STAT3 and T_h_17 immune response in the colonic mucosa. STAT3 activation induces pro-oncogenic inflammatory pathways and increases the permeability of mucosa. Although STAT3 activation is long-term and lasts for months, it highly increases the chance of getting a tumour as a result of chronic inflammation. Additionally, STAT3 activation promotes the accumulation of tumour regulatory T-cell (Tregs) and blocks the generation of anti-tumour immune responses, which give an adverse effect to the body. This abnormal persistent STAT3 activation increases the cancer cell tolerance, prevents rejection of cancer by the immune system, reduces the effectiveness of immunotherapy and enhances the effectiveness of oncogenesis (10, 17, 44, 50, 51). Activated STAT3 predominantly detected in human cancers is constitutively activated and depicts its association with neoplasms (45). Patients with IBD tend to show STAT3 activation and a high level of T_h_17 cells and IL-17. The level of activated STAT3 in patients with IBD and dysplasia is different from patients with IBD and without dysplasia. Patients with IBD and dysplasia show a higher level of activated STAT3 compared to those without dysplasia. Simultaneously, the level of activated STAT3 increases together with the continuum of dysplasia to colitis-associated cancer (10, 47, 52). It is clear that *B. fragilis* can either be toxigenic or non-toxigenic; the latter does not activate STAT3 because it does not produce BFT. Therefore, NTBF does not contribute to colon cancer development, but ETBF does (48).

### Are Tregs, T_h_17 and IL-17 Good or Bad?

**Figure 1:**
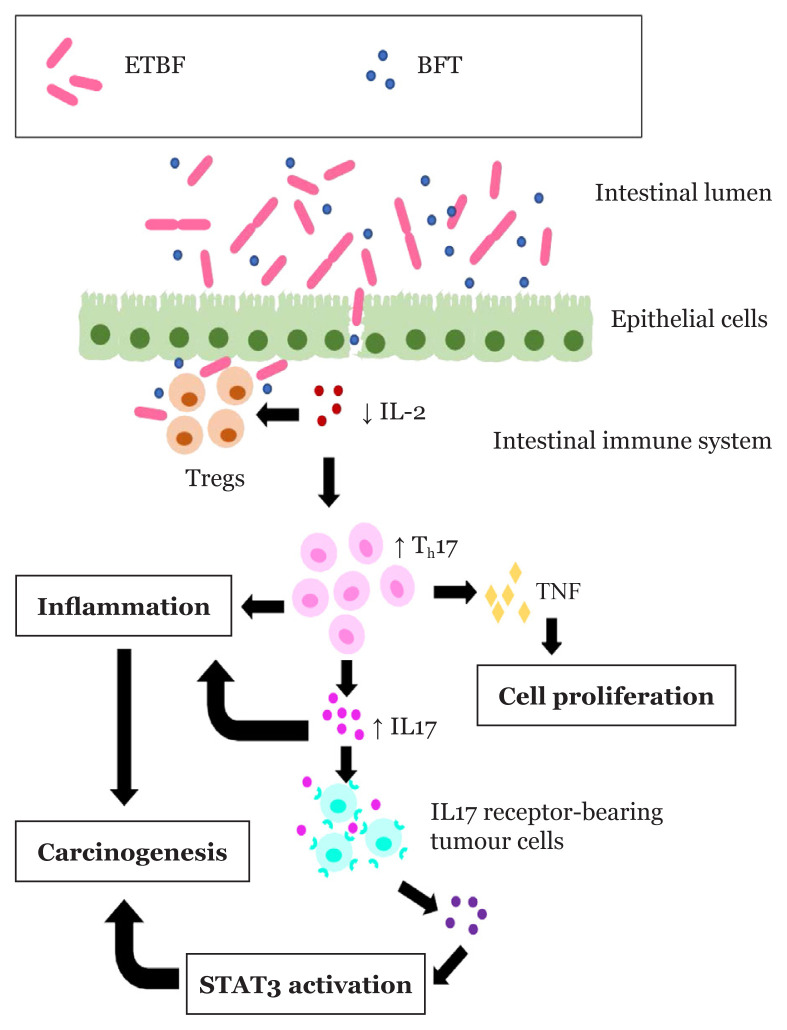
The mechanism of carcinogenesis through abnormal intestinal immune system

In a normal healthy condition, Tregs play an important role in inflammatory responses and intestinal immune homeostasis. They express high levels of IL-2 receptor and produce endogenous IL-2, which inhibits the production of IL-17. This process reduces intestinal inflammation and prevents carcinogenesis. However, when ETBF colonises a particular site of the colon, it produces a large amount of BFT damaging the intestinal mucosa to initiate ETBF-triggered colitis with the activation of the STAT3 pathway. This leads to direct contact between Tregs and ETBF and promotes Tregs activation. Activated Tregs lack the ability to produce endogenous IL-2 (53–56). Instead of producing endogenous IL-2, Tregs consume exogenous IL-2 for their survival. The consumption of exogenous IL-2 by Tregs reduces the levels of exogenous IL-2 and produces an environment that favours the growth of T_h_17 cells. As the levels of IL-2 drop, T_h_17 cells are no longer inhibited and undergo expansion to produce a large quantity of naïve T-cells. This naïve subset of T-cells then differentiates into T_h_17 cells in excess. This shows that colonisation of ETBF promotes the accumulation of both Tregs and T_h_17 cells (55–60). T_h_17 cells start to produce large amounts of cytokines, including TNF and IL-17. These cytokines promote cell survival and proliferation during injuries. Although T_h_17 cells heal an injured site, they turn into pathogenic T_h_17 cells when deregulated. These pathogenic T_h_17 cells initiate chronic inflammatory condition. IL-17 produced by pathogenic T_h_17 cells are involved in an early inflammatory stage of the injuries. It promotes tumour cell survival, proliferation, tumour neovascularisation and metastasis, which allow carcinogenesis (61–63). Additionally, tumour cells and fibroblasts are stimulated by IL-17 to produce high amounts of angiogenic factors for angiogenesis (64, 65). IL-17 can activate STAT3 pathway indirectly through IL-6 (49). When IL-17 binds to IL-17 receptor-bearing tumour cells, it stimulates IL-6 production that is highly important for STAT3 pathway activation as mentioned above. This STAT3 pathway activation contributes to several characteristics, such as cancer proliferation, anti-apoptosis and angiogenesis, that favour carcinogenesis in the colon (63, 66, 67). This shows that there is a relationship between STAT3 pathway and Tregs in contributing to CRC formation when ETBF is accumulating in the intestinal tract, as shown in Figure 1 (68). To some extent, STAT5 and STAT6 have been reported to be involved in inhibiting anti-tumour immunity. When all STAT3, 5 and 6 are activated together, it highly enhances the tumourigenesis effect (17).

### Cleavage of E-Cadherin Stimulate Cell Proliferation

Apart from inflammation, BFT alters the structure and function of colon epithelial cells by degrading E-cadherin (20). E-cadherin is a 120-kDa glycoprotein that is the major structural protein in zonula adherens and is also known to be a tumour suppressor and zonula adherence protein. This protein is responsible for the epithelial polarity. In normal conditions, the expression of E-cadherin is linked to cellular functions, including apoptosis and homotypic cell–cell adhesion (69–71). Unfortunately, when E-cadherin interacts with BFT in the intestinal epithelial cells, it degrades E-cadherin rapidly in an ATP-independent manner. This cleavage promotes colonic injury, inflammation and loss of membrane-association, resulting in morphological changes, and enhances cellular metastatic potential. It is proven that the cleavage of E-cadherin correlates with the changes of cell morphologies. Simultaneously, degradation of E-cadherin also promotes the binding of nuclear localisation of β-catenin and T-cell factor-dependent transcriptional activator (40, 57, 71). This binding promotes gene regulation and transcription. Additionally, β-catenin plays an important role in wingless and int (WNT) signalling pathway, promoting cell proliferation and epithelial-mesenchymal transition and enhancing the expression of proto-oncogene (20, 72). In primary colorectal tumours, cells in the centre of the tumour exhibit the presence of β-catenin and E-cadherin. However, when the cells move away from the centre of the tumour, they exhibit high amounts of nuclear β-catenin, and the junction of E-cadherin is lost (73).

E-cadherin plays an important role in maintaining the morphology of cells. There is a relationship with the E-cadherin and the apical F-actin ring of the intestinal epithelial cells’ secretion. When the loss of E-cadherin increases, the integrity of the apical F-actin is lost, resulting in the increase in cell volume and chloride secretion, and cell and epithelial barriers become more permeable (74, 75). This contributes to intestinal inflammation, diarrhoea and colon carcinogenesis.

### Alteration of the Signalling Pathway of Colorectal Cancer

**Figure 2:**
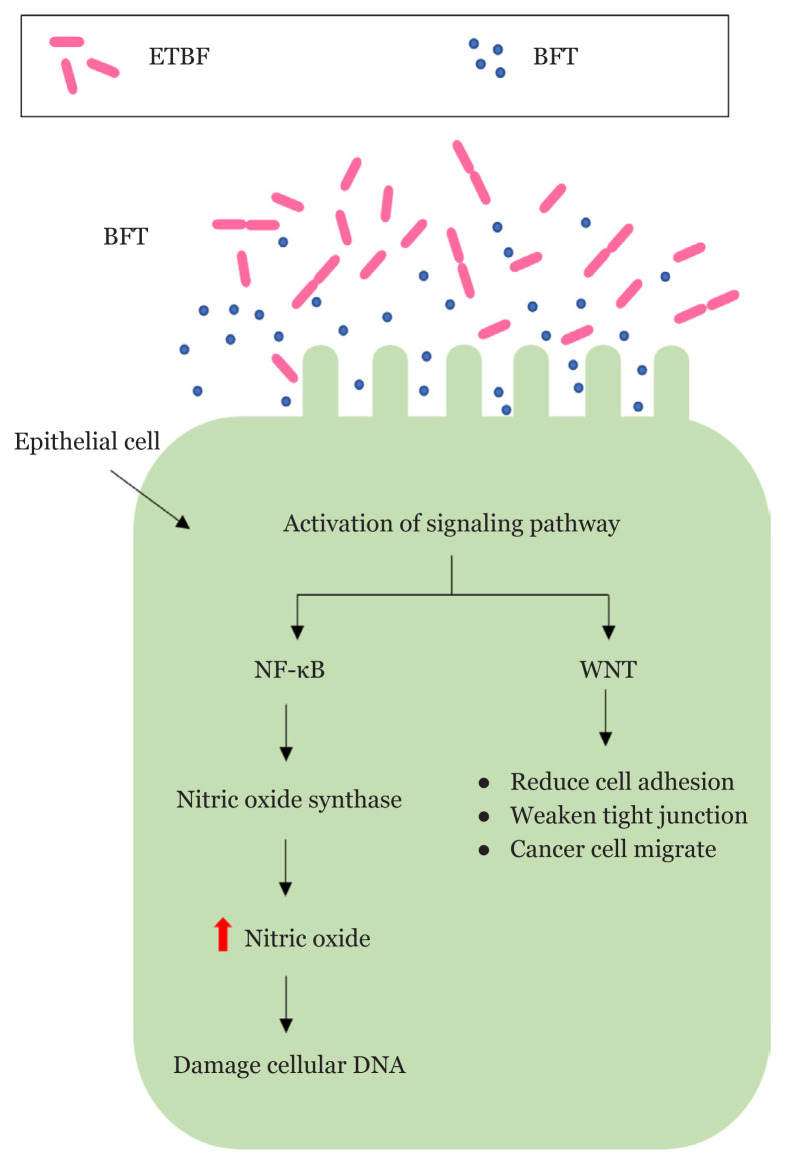
The role of the signalling pathways when epithelial cells contact BFT

BFT is involved in many colonic epithelial cell signal transductions. When BFT disturbs or activates the signalling pathway, it brings adverse effects to the body and can lead to colorectal tumourigenesis (Figure 2). The colonic epithelial cell signal transduction transpires through the nuclear factor kappa-light-chain-enhancer of activated B-cells (NF-κB), WNT and mitogen-activated protein kinase (MAPK) signalling pathways (76, 77). BFT can stimulate NF-κB pathway in the intestinal epithelial cells with the expression of heme oxygenase-1 (HO-1) and cytokines to induce mucosal inflammation. This pathway has the ability to enhance the survival of neoplastic cells by preventing them from undergoing apoptosis, leading to tumour formation (78, 79). Furthermore, in Figure 2, it shows that when NF-κB of intestinal epithelial cells is activated for a long time, it induces the activity of nitric oxide synthase that breaks down L-arginine to produce nitric oxide, which can damage cellular DNA (72, 80). WNT signalling pathway is important to maintain the structures of the intestinal epithelium. However, WNT signalling pathway contributes negatively and affects cells which are extremely important for colorectal carcinogenesis and progression (81). As WNT signalling pathway is activated, it weakens tight junctions and reduces cellular adhesion. This allows the cancer cells to undergo migration and metastasis. Hence, cancerous cells can migrate to another organs (82).

**Figure 3:**
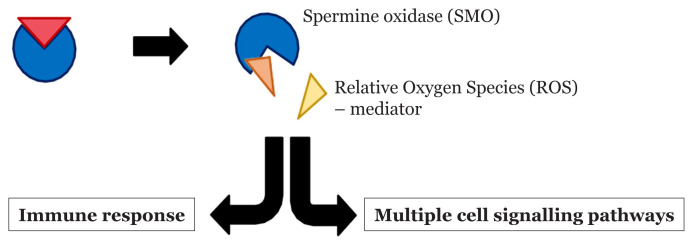
Normal condition of the SMO and ROS that helps in immune response and cell signalling pathways

**Figure 4:**
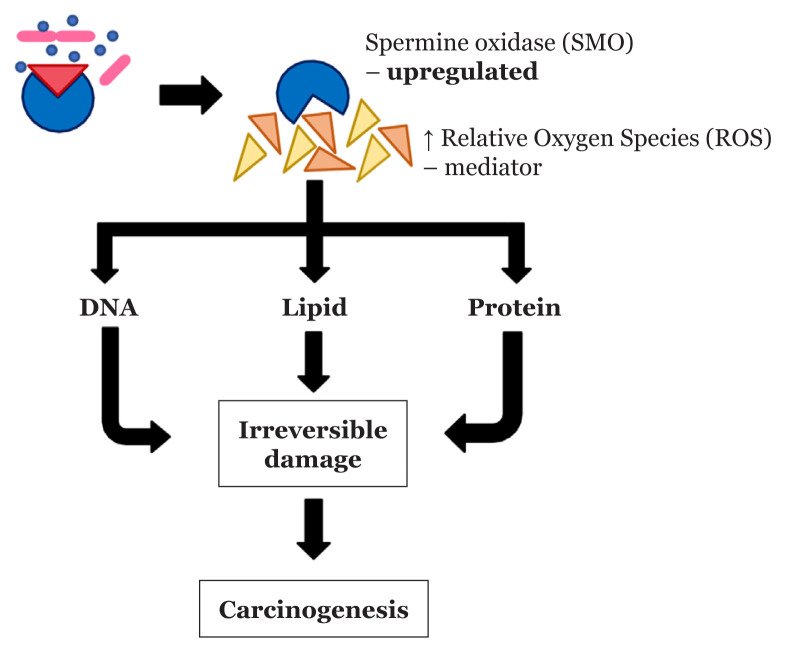
The adverse effect of SMO contacted BFT

Spermine oxidase is a catabolic enzyme that increases ROS, which can be upregulated by BFT (83). In normal conditions (Figure 3), ROS acts as an important mediator in multiple cell signalling pathways and immune response that is produced naturally within biological systems. It consists of superoxide, hydroxyl radical and hydrogen peroxide. However, as the amount of ROS becomes excessive, it imparts negative effects in the disruption of redox homeostasis (Figure 4). This excessive ROS induces oxidative stress. It oxidises cellular components, including DNA, lipids and proteins, within the cells. Once the cellular components are oxidised, it generates irreversible damage to host cells. Additionally, ROS plays an important role in the survival of cancer cells, enhancing the effectiveness of carcinogenesis and aggravating cancer formed in the body (84–86).

### Familial Adenomatous Polyposis

The combination of both genetic and environmental factors contributes to CRC formation. It is estimated that > 35% of CRC development is due to genetic predisposition, wherein nearly 1% of all CRCs are attributed to familial adenomatous polyposis (FAP) (87, 88). FAP is an autosomal dominant inherited disorder that describes the development of numerous colorectal adenomatous polyps. These polyps are able to develop in the teenager’s colon. Meanwhile, the number of polyps formed in the colon depends on the age of a person, which means the number of polyps is directly proportional to the age of a person. If these polyps are not removed from the colon, they may transform from benign to malignant, developing CRC. The source of FAP disease is mainly due to germline mutation in the *adenomatous polyposis coli* (*APC*) gene (89–91). This *APC* mutation occurs due to frameshifts, insertions or deletions that may introduce a premature stop codon during the halfway through the transcription process. These early-introduced premature stop codons in the gene sequence lead to incomplete/truncated *APC* protein formation. Thus, the normal function of *APC* protein is lost, eventually facilitating carcinogenesis (92). Additionally, germline mutations along with somatic mutations of the normal allele or loss of the normal allele lead to inactivation of *APC*. Once *APC* is inactivated, it precisely commences carcinogenesis (93). In normal conditions, *APC* pathway acts as a gatekeeper, controlling a part of WNT signalling pathway. Unfortunately, when *APC* is mutated, the function of *APC* pathway is lost or inactivated. This inactivation of the *APC* pathway results in the activation of WNT signalling pathway. This characteristic is mainly found in CRC (94, 95). Moreover, *APC* mutation has the ability to alter bacteria–host epithelial interaction, where it allows the bacteria to attach onto the mucosa (96). If a person has the *APC*-mutated gene and is exposed to ETBF, the chances of developing CRC are high. Concurrently, high amount of tumour load is displayed in the person’s colon (97).

## Conclusion

The human gastrointestinal tract contains its own bacterial flora that benefit humans daily. *B. fragilis* is one of them and consists of two classes, namely NTBF and ETBF. The differences between both the classes is the presence of *bft*. ETBF is able to produce BFT that can disrupt the intestinal environment and promotes inflammation. Simultaneously, BFT degrade E-cadherin and causes inflammation. IBD is a chronic intestinal inflammation associated with ETBF and can induce CRC. However, patients with CD have lower risk of developing CRC as compared to those with UC. Patients with IBD exhibit STAT3 activation due to the stimulation of immune response that favours T_h_17 cell generation. As the levels of T_h_17 cell increase, it brings a huge disadvantage to the intestinal tract due to the production of IL-17. Furthermore, IL-17 stimulates the production of IL-6 that is required to activate STAT3. This indicates that the STAT3 pathway activates for a long time. Long-term STAT3 activation blocks anti-tumour immune response, which supports the growth of cancer cells. Thus, STAT3, T_h_17 and IL-17 are highly important in carcinogenesis. Concurrently, the production of proinflammatory cytokines at the site of inflammation triggers the production of COX-2 enzymes that release PGE2. COX-2 is also known for its carcinogenic abilities due to the production of PGE2 that controls cell proliferation. Additionally, BFT affects signal transductions, such as WNT, NF-κB and MAPK signalling pathways, and induces tumourigenesis. Considering that BFT induces inflammation, activates STAT3 and alters signalling pathways, it can be concluded that BFT produced by ETBF plays an important role in colon carcinogenesis.
